# Supplementary material for: Consensus on the Best Practice Guidelines for Psychomotor Intervention in Preschool Children with Autism Spectrum Disorder
Source: Children (Basel). 2022 Nov 19;9(11):1778. doi: 10.3390/children9111778 (PMC9688804; doi:10.3390/children9111778)
Supplement: Supplementary file 1 [file children-09-01778-s001.zip › children-1975188-supplementary.pdf]

Supplementary data

Table S1. Statements rated in round 1 and 2.

**Statements of round 1**	% agree ment	From round 1 to round 2	**Statements of round 2**	% agree ment	
**A. Intervention source**					
1. Psychomotor intervention is a therapy that facilitates opportunities for play and movement-based experiences for the child to explore, experience and feel competent in bodily interaction with context, in the educational and therapeutic approaches.	97.1	*	1. Psychomotor intervention facilitates play opportunities and movement-based experiences for the child to explore, experience and feel competent in the bodily interaction with the context, in educational and therapeutic approaches.	95.7	✓
**B. General setting**					
2. The intervention takes place inside the classroom with other children.	42.9	×			
3. The intervention takes place in the playground, with other children.	45.7	×			
4. The intervention takes place in a gymnasium/school room defined for this purpose.	74.3	×			
5. The intervention takes place in a therapeutic room of a clinic/hospital/institution.	74.3	×			
6. Intervention takes place at home (individual sessions).	57.1	×			
			2. The psychomotor intervention should take place in a room intended for sessions, either in the school, clinical or in a community context.	65.2	×

			3. Psychomotor intervention can take place in different spaces (home, swimming pool, equestrian center, outdoor spaces) depending on the child's needs and objectives.	95.7	✓
			4. Psychomotor intervention can occur individually or in groups, where individual interventions allow the strengthening of the therapeutic relationship and the reinforcement of skills with individualized strategies.	100.0	✓
**C.**			**Venue characteristics**		
7. The space should guarantee the child's safety and autonomy.	100.0	✓	5. The space should guarantee the child's safety and autonomy.	100.0	✓
8. The space should maintain the same general structure throughout the intervention process.	51.4	×			
9. The space should have a rest area and a large area that allows for whole body movement.	82.9	*	6. The space should be large, allowing whole body movement and tranquility/rest moments.	87.0	✓
10. Space may include a table and two small chairs.	88.6	✓	7. Space may include a small table and chairs.	87.0	✓
11. The space should allow the control of the sensorial stimuli and the selection of those that allow the best sensorial regulation of the child in the activities.	88.6	✓	8. Space should allow control of sensory stimuli in order that sensory regulation of the child in the activities is possible.	87.0	✓
**D.**			**Length of sessions**		
12. The session lasts 45 to 50 minutes (individual session)	71.4	*	9. Session’s length (individual or group) should be approximately of 45 minutes.	82.6	✓
13. The session lasts 60 minutes (group session)	57.1	×			
**E.**			**Frequency of sessions**		
14. Frequency of at least twice a week (individual session)	68.6	*	10. Sessions (individual or in groups) should have a weekly frequency.	65.2	×
15. Weekly frequency (group session)	54.3	×			

**F. Duration of the intervention**					
16. There is no standard regarding the minimum or maximum number of sessions.	54.3	*	11. The duration of the intervention depends on the child's profile, the work carried out with the family and the school context, and the articulation between stakeholders and resources.	100.0	✓
**G. Change mechanisms or theories of change**					
17. The preschool period is a sensitive period regarding environmental stimuli related to motor and social development.	100.0	✓	12. The preschool period is a sensitive period regarding environmental stimuli related to motor and social development.	95.7	✓
18. Neuroplasticity is stimulated by the stimuli of involvement characterized by a diversity of opportunities for action intentionally induced to awaken the child's initiative, a sense of agency and autonomy	94.3	*	13. Neuroplasticity is stimulated by the diversity of actions and opportunities within the context that are intentionally induced to stimulate the child's initiative, agency and autonomy.	91.3	✓
19. The child's motivations (interests) are the starting point for the development of competences.	97.1	✓	14. The child's motivations (interests) are the starting point for the development of competences.	100.0	✓
20. The playfulness in the psychomotor therapist's approach promotes active, interactive and pleasurable learning.	100.0	✓	15. Playfulness, in the psychomotor therapist's approach, which promotes active, interactive, and pleasurable learning.	100.0	✓
21 - The therapeutic relationship should be guided by active listening, empathy, authenticity and trust.	100.0	✓	16. The therapeutic relationship should be guided by active listening, empathy, authenticity, and trust.	100.0	✓
22 - Safety is provided by a structured space and time, and by a welcoming and affectionate climate.	94.3	✓	17. Safety is provided by a structured space and time, and by a welcoming and affectionate climate.	95.7	✓
23. Sensorimotor experiences promote sensory information processing and processing related to planning, learning and control.	94.3	✓	18. Sensorimotor experiences promote sensory information processing and processing related to planning, learning and control.	100.0	✓
24. Motor behavior is important for the development of adaptive, cognitive, social, and linguistic skills.	94.3	*	19. Motor behavior is important for the development of adaptive, cognitive, socio- emotional and linguistic skills.	91.3	✓

25. The bodily movement produces physiological and emotional responses that are regulated according to the interaction with the social and physical environment and is systematized in the implicit memory responsible for the pleasure in the action and in the relationship with the other.	97.1	✓	20. The bodily movement produces physiological and emotional responses that are regulated according to the interaction with the social and physical environment and is systematized in the implicit memory responsible for the pleasure in action and in the relationship with the other.	95.7	✓
26. Awareness of the body (and self) influences the ability to feel and understand the emotional state of the other and, therefore, influences interpersonal relationships.	100.0	✓	21. Body (and self) awareness influences the ability to feel and understand the emotional state of the other and, therefore, influences interpersonal relationships.	100.0	✓
27. Gesture/motor imitation (resulting from the functioning of the mirror neuron system) is a form of social learning that underlies the development of more complex social skills.	94.3	✓	22. Gesture/motor imitation (resulting from the functioning of the mirror neuron system) is a form of social learning that underlies the development of more complex social skills.	82.6	✓
28. The use of expression-oriented activities/strategies and action-oriented activities/strategies provides a comprehensive and integrated response to a child's development.	94.3	✓	23. The use of expression-oriented activities/strategies and action-oriented activities/strategies provides a comprehensive and integrated response to a child's development.	95.7	✓
29. One-to-one intervention favors intrapersonal development more.	77.1	✓	24. One-to-one intervention favors intrapersonal development more.	87.0	✓
30. Group intervention favors interpersonal development.	82.9	✓	25. Group intervention favors interpersonal development.	82.6	✓
	H.	**Change techniques**			
31. The techniques applied in the age group from 3 to 6 years-old in the context of a gymnasium or therapeutic room should be promoted by different types of play, selected according to the individual characteristics and interests of the child, as well as the objective of the intervention.	97.1	✓	26. The techniques applied in the age group from 3 to 6 years-old in the context of a gymnasium or therapeutic room should be promoted by different types of play, selected according to the individual characteristics and interests of the child, as well as the objective of the intervention.	95.7	✓
32. The type of play games in the first intervention sessions should be based on the child's spontaneity and/or interests.	88.6	✓	27. The type of play games in the first intervention sessions should be based on the child's spontaneity and/or interests.	95.7	✓

33. In movement play, free exploration of psychomotricity materials intentionally made available in the room, should be privileged.	74.3	*	28. In movement play, exploration of free movement should be promoted, with or without materials intentionally made available.	91.3	✓
34. In movement play, a sequence of movements organized in the form of a pathway or circuit should be promoted.	65.7	*	29. In movement play, structured opportunities for performing bodily actions (e.g., using pathways/circuits) should be promoted.	87.0	✓
36. In movement play, opportunities for physical contact play (e.g., hit and run, catch) should be promoted.	88.6	✓	30. In movement play, opportunities for physical contact play (e.g., hit and run, catch) should be promoted.	91.3	✓
35. In movement play (fine subtype), drawing, painting, modeling, fine manipulation and writing materials should be provided in different axes and spatial dimensions.	85.7	✓	31. In movement play (fine subtype), drawing, painting, modeling, fine manipulation and writing materials should be provided in different axes and spatial dimensions.	95.7	✓
37. In movement games, massage and imitation of simple movements involved in a story or song can be used to provide the perception of the contrast between contraction and relaxation.	82.9	✓	32. In movement games, massage and imitation of simple movements involved in a story or song can be used to provide the perception of the contrast between contraction and relaxation.	95.7	✓
38. In expressive play, different music with rhythm involving different sequences of movements should be used.	88.6	*	33. In expressive play, different music and rhythms should be used, creating sequences of movements.	95.7	✓
39. In expressive play, different types of displacements, positions and body postures varying between states of movement and immobility should be facilitated.	97.1	✓	34. In expressive play, different types of movements, positions and body postures varying between states of movement and immobility should be facilitated.	95.7	✓
40. In expressive play, opportunities for dancing or free movement according to the rhythm of music should be promoted.	91.4	✓	35. In expressive play, opportunities for dancing or free movement according to the rhythm of music should be promoted.	91.3	✓
41. In expressive play, sounds, songs, words or narratives, tales or stories should be used.	91.4	✓	36. In expressive play, sounds, songs, words or narratives, tales or stories should be used	91.3	✓
42. In sociodramatic imagination play, social roles, animals, and everyday actions should be played.	91.4	✓	37. In sociodramatic imagination play, social roles, animals, and everyday actions should be played.	91.3	✓

43. In symbolic imagination play, objects, actions, or ideas that symbolize something should be used.	97.1	✓	38. In symbolic imagination play, objects, actions, or ideas that symbolize something should be used.	95.7	✓
44. In games with organic rules (i.e., car racing), the child should be given the opportunity to invent rules in play situations, negotiate and challenge with other child.	97.1	✓	39. Games with organic rules (i.e., car racing), the child should be given the opportunity to invent rules in play situations, negotiate and challenge with other child.	91.3	✓
45. In games with conventional rules (e.g., playing football) knowledge of rules should be promoted to "know-how" that enable the mobilization of skills of perception, memorization, planning, organization, sequencing and association.	94.3	✓	40. In conventional rules games (i.e., playing football), knowledge of rules for "know what to do" should be promoted to enable the mobilization of skills of perception, memorization, planning, organization, sequencing and association.	95.7	✓
46. Games with rules (organic and conventional) should be promoted in pairs and/or in groups for the assimilation of rules traditionally/universally known.	91.4	*	41. Universal games in pairs and in groups should be promoted in rules games.	91.3	✓
47. In constructive exploration play construction materials should be made available.	91.4	✓	42. In constructive exploration play (i.e., building a tower), construction materials should be made available.	95.7	✓
48. In sensory exploration play, materials with different sensory properties (visual, auditory, tactile, proprioceptive, vestibular) should be made available.	91.4	✓	43. In sensory exploration play, materials with different sensory properties (visual, auditory, tactile, proprioceptive, vestibular) should be made available.	95.7	✓
49. In sensory exploration play, experiences of therapeutic/affective touch, breathing, body containment and passive mobilization should be promoted using mediation materials.	94.3	✓	44. In sensory exploration play, experiences of therapeutic/affective touch, breathing, body restraint and passive mobilization should be promoted using mediation materials.	95.7	✓
	I.	**Session content**			
50. All sessions should begin and end with a start and end point that is consistently repeated in all sessions.	88.6	✓	45. All sessions should begin and end with a start and routine that is congruently repeated in all sessions.	73.9	×
51. Each session contains a core part focused on developing at least one of the following goals: sensorimotor exploration, body	88.6	✓	46. Each session contains a core part focused on developing at least one of the following goals: sensorimotor exploration, body awareness, emotional and	82.6	✓

awareness, emotional and behavioral regulation, social interaction, and communication.			behavioral regulation, social interaction, and communication.		
52. The review of the experiences/tasks that took place in the session should be carried out by the child or by the psychomotor therapist to promote the representation of the situations experienced during the session and allow an analysis about the child's learning.	88.6	✓	47. The review of the experiences/tasks that took place in the session should be carried out by the child or by the psychomotor therapist to promote the representation of the situations experienced during the session and allow an analysis about the child's learning.	82.6	✓
		**J.**	**Sequencing of sessions**		
53. At the end of the sessions, the psychomotor therapist should give positive feedback to the parents or educators about the child behavior observations and progressions.	82.9	↓			
54. The following session should be a continuation of the previous session in terms of objectives.	71.4	×			
55. In all sessions, an introduction should be carried out with a child at the beginning of the session, about what happened in the previous session and at the end of the sessions about what happened in that session.	51.4	×			
		**K.**	**Materials**		
56. Materials should be tidy when the child enters the session, preferably not visible.	31.4	×			
57. Materials should be prepared in advance according to planning and made available as activities take place.	68.6	×			
58. Materials should be carefully selected according to their properties and possibility of action.	85.7	✓	48. Materials should be carefully selected according to their properties and possibilities of action.	91.3	✓
59. Materials should be put away from the child whenever the session ends or when the game is predicted to be over, opening and closing communication loops.	91.4	✓	49. Materials should be put away from the child whenever the session ends or when the game is predicted to be over, opening and closing communication loops.	87.0	✓

60. Large materials (e.g., tents, tunnels, foam blocks, large balls, balloons, mattresses, trampoline, swing) are used to facilitate social interaction.	68.6	×			
61. Small materials (e.g., fitting materials, threads, tactile sensory exploration, bows) are used to promote individual activities.	40.0	×			
**L.**	**Activities during the sessions**				
62. Activities should be unstructured if expression is to be facilitated.	60.0	×			
63. Activities should be structured when it is intended to develop a specific competence.	54.3	×			
64. All sessions should involve the child in choosing activities or activity components (game, material or space), according to their interests.	68.6	×			
65. The transition between activities should be predictable through the use of a sound, song, word, rhyme, story, specific placement in a room space, specific group arrangement (e.g., in a circle) or predetermined sequence of activities.	71.4	×			
**M.**	**Strategies during the sessions**				
66. The child's interests, curiosity, desire for exploration and initiatives should be followed to promote active engagement and learning.	100.0	✓	50. The child's interests, curiosity, desire for exploration and initiatives should be followed to promote active engagement and learning.	100.0	✓
67. Environmental manipulation through the organization of space, the selection of materials and the positioning of the psychomotor therapist's body should guide the exploration of the child's possibilities of action, introducing or removing elements.	94.3	✓	51. Environmental manipulation through the organization of space, the selection of materials and the positioning of the psychomotor therapist's body should guide the exploration of the child's possibilities of action, introducing or removing elements.	100.0	✓

68. Verbal instruction should be clear, simple/short and directive and can be associated with gestures or demonstration.	94.3	✓	52. Verbal instruction should be clear, simple/short and directive and can be associated with gestures or demonstration.	100.0	✓
69. Visual or gestural cues (or prompts) should be used when there is a need to guide the child's response in accordance with the objective, during instruction or execution of the activity.	85.7	✓	53. Visual or gestural prompts should be used when there is a need to guide the child's response in accordance with the objective, during the instruction or execution of the activity.	100.0	✓
70. Self-instruction should be used for the child to verbalize a sequence of actions for the psychomotor therapist verify that the child has understood the instruction.	80.0	✓	54. Self-instruction should be used for the child to verbalize a sequence of actions for the psychomotor therapist to verify that the child has understood the instruction.	87.0	✓
71. The demonstration should accompany the instruction, and can be carried out by the psychomotor therapist, by peer or with the physical manipulation of the child him/herself.	91.4	✓	55. The demonstration should accompany the instruction, and can be performed by the psychomotor therapist, by peer or with the physical manipulation of the child him/herself.	87.0	✓
72. Presentation and manipulation of materials should be done intentionally at eye level of the child.	82.9	✓	56. Presentation and handling of materials should be done intentionally at eye level of the child.	87.0	✓
73. The presentation of game materials by boxes, bags or drawers that open and close, "one at a time", should be a strategy used when there are difficulties regulating attention or behavior.	85.7	✓	57. The presentation of game materials by boxes, bags or drawers that open and close "one at a time" should be a strategy used when there are difficulties in regulating attention or behavior.	82.6	✓
74. Imitation should be carried out to arouse and facilitate social interaction and the learning of social behaviors (modelling), gestures, motor actions, facial expressions, sounds (including using mirrors and images).	94.3	✓	58. Imitation should be carried out to arouse and facilitate social interaction and the learning of social behaviors (modelling), gestures, motor actions, facial expressions, sounds (including using the mirror and images).	87.0	✓
75. Augmentative or alternative communication systems (e.g., PECS, pictographic images) should be used to facilitate communication.	77.1	✓	59. Augmentative or alternative communication systems (e.g., PECS, pictographic images) should be used to facilitate communication.	65.2	×

76. The total or partial physical and verbal prompts that leads to the achievement of the objective in the activity should evolve from a greater to a lesser intensity.	94.3	✓	60. The total or partial physical and verbal prompts that leads to the achievement of an objective in the activity should evolve from a greater to a lesser intensity.	95.7	✓
77. Repetition of activities should be promoted to improve and consolidate skills acquisition.	82.9	✓	61. Repetition of activities should be promoted to improve and consolidate skills acquisition.	69.6	×
78. The analysis or task decomposition in a sequential way (task analysis) should lead to autonomy and the perception of self-competence in the activities.	94.3	✓	62. The analysis or task decomposition in a sequential way should lead to autonomy and the perception of self-competence in the activities.	87.0	✓
79. The adequacy of the level of complexity of the activities should favor the child's motivation and involvement.	94.3	✓	63. The adequacy of the level of complexity of the activities should favor the child's motivation and involvement.	91.3	✓
80. The psychomotor therapist should create challenges between the child and what he/she wants to do to promote the children interaction/communication, when contextual conditions and a trusting therapeutic relationship exists.	88.6	✓	64. The psychomotor therapist should create challenges between the child and what he/she wants to do to promote the children interaction/communication, when contextual conditions and a trusting therapeutic relationship exists.	95.7	✓
81. Anticipating what will happen through planning the session structure with the child and establishing routine moments should happen to help the child manage his/her emotions and behaviors, through drawing, images, verbalization, repeated actions and definition of spaces and materials.	88.6	✓	65. Anticipating what will happen through the planning the session structure and the establishment of routine moments, help the child manage his/her emotions and behaviors, through drawing, images, verbalization, repeated actions and definition of spaces and materials.	95.7	✓
82. The description of child' observable emotions and behaviors should be used whenever there is a need to help the child to regulate him/herself.	97.1	✓	66. The description of the child observable emotions and behaviors should be used whenever there is a need to help the child to regulate him/herself.	100.0	✓
83. The therapist's tonic receptiveness should induce the child's emotional safety, interaction, and communication through the psychomotor therapist's posture, looking at the child's eye level, using synchrony of verbal and non-verbal communication and voice tone.	97.1	✓	67. The therapist's tonic receptiveness should induce the child's emotional safety, interaction, and communication through the psychomotor therapist's posture, looking at the child's eye level, using synchrony of verbal and non-verbal communication and voice tone.	100.0	✓

84. Affective touch, restraint and holding should be used, if the child is receptive, to help regulate his/her behavior and attention.	100.0	✓	68. Affective touch, restraint and holding should be used if the child is receptive, to help regulate his/her behavior and attention.	91.3	✓
85. Questioning should occur for the child to perceive and reflect on his/her feelings, behaviors, and proficiency in tasks.	85.7	✓	69. Questioning should occur for the child to perceive and reflect on his/her feelings, behaviors, and proficiency in tasks.	87.0	✓
86. Feedback should be positive (i.e., focused on the child's promising/emerging learning and behaviors), continuous and consistent/coherent, centered on the outcome of the action and it can be verbal, gestural and/or visual (with photographs and videos).	91.4	✓	70. Feedback should be positive (i.e., focused on the child's promising/emerging learnings and behaviors), continuous and consistent/coherent, centered on the outcome of the action and it can be verbal, gestural and/or visual (with photographs and videos).	91.3	✓
87. Reinforcement should be positive and evolve reducing progressively its frequency (from some to no frequency) to reinforce that a desired behavior happens again, it can be natural (intrinsic) or provided through praise or the exchange of rewards (activities or objects of interest, token system/ tokens).	77.1	✓	71. Reinforcement should be positive and evolve reducing progressively its frequency (from some to no frequency) to reinforce that a desired behavior happens again, it can be natural (intrinsic) or provided through praise or the exchange of rewards (activities or objects of interest, token system/ tokens).	87.0	✓
**N. Methods for generalization**					
88. The generalization of learning should be promoted in the sessions, through the repetition of activities in which small changes are introduced in terms of materials, speed, space, duration, therapist's approach, among others.	88.6	✓	72. The generalization of learning should be promoted in the sessions, through the repetition of activities in which small changes are introduced in terms of materials, speed, space, duration, therapist's approach, among others.	87.0	✓
89. Exposure to social situations in a real context as a technique that can promote the generalization of skills should occur when the psychomotor therapist observes in a one-to-one relationship and checks that the child is prepared to mobilize skills in a real context.	91.4	✓	73. Exposure to social situations in a real context as a technique that can promote the generalization of competences should occur when the psychomotor therapist observes in a one-to-one relationship and checks that the child is prepared to mobilize competences in a real context.	91.3	✓
**O. Methods for checking fidelity of delivery**					

90. All people involved with the child (therapists, caregivers, educators, etc.) should base their intervention on the single intervention plan and review it every 3 months.	54.3	✓	74. All those involved with the child (therapists, caregivers, educators, etc.) should base their intervention on a collaborative intervention plan.	87.0	✓
91. Assessment of the child should occur at the beginning, middle (quarterly to semester) and end of the intervention.	65.7	*	75. Formal assessment of child should take place at the beginning of the intervention and be reviewed periodically.	82.6	✓
92. All sessions should be planned with operational objectives with success criteria.	60.0	×			
93. Priority objectives are selected according to the expected time of intervention and according to participants' feedback.	80.0	✓	76. Priority objectives are selected according to the expected time of intervention and according to stakeholder feedback.	73.9	×
95. Planning should foresee and record the methodologies' adaptation to interests, needs, abilities, difficulties, joint attention time, emotional and behavioral state, and context.	94.3	↑	77. Planning should foresee and record the adaptations of methodologies to interests, needs, abilities, difficulties, joint attention time, emotional and behavioral state, and context.	91.3	✓
94. In all sessions, a brief record should be made about the description of activities carried out, qualification of acquisitions/progress and observations of behavior and relationship and sharing of relevant information with parents/educators.	94.3	✓	78. In all sessions, a brief record should be made about description of activities carried out, qualification of acquisitions/progress and observations of behavior and relationship and/or sharing of relevant information with parents/educators.	91.3	✓
**P.**	**Methods for checking social validity**				
			79. At the end of the sessions it is recommended to give feedback on the child's performance.	82.6	✓
102. Regular moments of formal (monthly) or informal (weekly) conversations about understanding behavior, context, strategies, and activities for home/school inserted in family or educational routines should be promoted.	94.3	↑	80. Regular moments of (informal) information sharing on understanding behavior, context, strategies, and activities for home/school should be promoted as part of family or educational routines.	91.3	✓
96. Periodic meetings (monthly to quarterly) should be promoted with parents to analyze the intervention plan, review	91.4	✓	81. Periodic meetings (monthly to quarterly) should be promoted with parents to analyze the intervention plan,	91.3	✓

the objectives, identify progressions and make the necessary adjustments.			review the objectives, identify progressions and make the necessary adjustments.		
		**Q. Participants**			
97. All child aged 3 to 6 years (with or without ASD) can benefit from psychomotor intervention.	94.3	✓	82. All child aged 3 to 6 years (with or without ASD) can benefit from psychomotor intervention.	91.3	✓
101. Parents and educators/therapists may observe some intervention sessions with the child.	85.7	✓	83. Parents and educators/therapists may observe some intervention sessions with the child.	82.6	✓
110. Another adult (educator, family member, coach) who actively participates in the session should be attentive and supportive of the child's needs.	97.1	↑	84. Parents and educators/coaches can actively participate in the session, adopting an attitude of listening and supporting the child's needs.	82.6	✓
98. In the group session reference peers may be invited based on specific criteria and allowing different pairing over time.	85.7	↓	85. In the group session reference peers may be invited based on specific criteria and allowing different pairing over time.	82.6	✓
		**R. Therapists delivering the sessions**			
99. The psychomotor therapist should guarantee the commitment and professional competence in the monitoring of intervention centered on the child and the family.	97.1	✓	86. The psychomotor therapist should guarantee the commitment and professional competence in the monitoring of intervention centered on the child and the family.	100.0	✓
100. The psychomotor therapist should have to supervision/tutoring and continuous training, based on scientific evidence.	100.0	✓	87. T The psychomotor therapist should have supervision/tutoring and continuous training, based on scientific evidence.	100.0	✓
		S. **Intended facilitation style**			
103. The psychomotor therapist should provide conditions for the child to express and put into practice their abilities (strengths) to reduce their difficulties (weaknesses).	97.1	✓	88. The psychomotor therapist should provide conditions for the child to express and put into practice their abilities (strengths) to reduce their difficulties (weaknesses).	95.7	✓
104. The psychomotor therapist should be an authority figure and a game partner when intentionally intending to set limits or facilitate expansion/expression, respectively.	91.4	✓	89. The psychomotor therapist should be an authority figure and a gaming partner when intentionally intending	91.3	✓

			to set boundaries or facilitate expansion/expression, respectively.		
105. The psychomotor therapist should be active in observing or participating with the child.	94.3	✓	90. The psychomotor therapist should be active in observing or participating with the child.	95.7	✓
106. The psychomotor therapist's approach should be directive or non-directive according to the child's characteristics and therapeutic goals.	94.3	✓	91. The psychomotor therapist's approach should be directive or non-directive according to the characteristics of the child and the therapeutic goals.	95.7	✓
107. The psychomotor therapist should establish an authentic and secure therapeutic relationship.	100.0	✓	92. The psychomotor therapist should establish an authentic and safe therapeutic relationship.	95.7	✓
108. The limits/rules actively defined by the group should be known for all the elements of the group, whenever necessary.	94.3	✓	93. The limits/rules actively defined by the group should be known for all the elements of the group, whenever necessary.	95.7	✓
109. The facilitation of group activities should provide for the participation of all group elements, making the necessary adaptations so that all children can demonstrate their abilities and welcoming different forms of performance.	91.4	✓	94. The facilitation of group activities should provide for the participation of all group elements, making the necessary adaptations so that all children can demonstrate their abilities and welcoming different forms of performance.	95.7	✓
111. Another adult who passively participates in the session should observe passively without interfering.	71.4	×			

Legend of symbols: ✓ - statements not reformulated; *- statements reformulated; × - statements excluded; ↓ - statements moved to the below sections; ↑ - statements moved to the above sections
